# Alpine Meadow Habitat Is Associated with Characteristic Flavor Formation in Bayinbuluke Sheep Meat Through Metabolic Reprogramming

**DOI:** 10.3390/foods15142515

**Published:** 2026-07-16

**Authors:** Yaling Yang, Wujun Liu, Hang Cao

**Affiliations:** Department of Animal Science, Xinjiang Agricultural University, Urumqi 830052, China; yangyaling3141@163.com (Y.Y.); lwj_ws@163.com (W.L.)

**Keywords:** Bayinbuluke sheep, high-altitude adaptation, flavor precursors, multi-omics, volatilomics

## Abstract

This study elucidates the biochemical mechanisms by which extreme high-altitude environments are associated with the remodeling of the skeletal muscle flavor profile in sheep. Using Bayinbuluke (high-altitude) and Turpan Black (low-altitude) sheep, we integrated volatilomics, metabolomics, transcriptomics, and proteomics to map flavor precursor networks. Odor activity value analysis revealed that high-altitude meat exhibited sub-threshold suppression of animal off-flavors, such as p-cresol, while significantly amplifying premium fruity esters. Targeted metabolomics attributed this sensory inversion to a structural shift in the precursor pool, specifically the enrichment of highly reactive polyunsaturated fatty acids, L-cysteine, and rhamnose. Multi-omics integration demonstrated that cold and hypoxic stresses are associated with metabolic reprogramming. The synergistic downregulation of the *GPAT3* gene and APOC3 protein was associated with redirected lipid flux, suggesting a mechanism for functional fatty acid accumulation. Simultaneously, the pronounced upregulation of the *CTH* gene, potentially driven by antioxidant defense needs, coincided with substantial L-cysteine accumulation. Correlation networks suggested that this precursor shift is negatively correlated with off-flavor generation, possibly via competitive thermal degradation. In conclusion, extreme ecological stress is associated with convergent metabolic reprogramming, contributing to the reconstruction of the flavor precursor pool to alter the characteristic lipid aroma of plateau meat. While these findings provide a comprehensive structural blueprint of plateau meat characteristics, the proposed mechanisms remain hypothetical and warrant future functional and sensory validation.

## 1. Introduction

Sheep meat flavor is a core eating quality trait determining consumer acceptance [1]. Historically, research on its formation mechanisms has predominantly focused on dietary transitions [2,3] or conventional postmortem aging processes [4,5]. However, the remodeling effect of geographical environments, particularly extreme altitudinal gradients, on the skeletal muscle flavor phenotypes of ruminants remains rarely reported in a systematic manner. Xinjiang, a typical transitional zone intersecting inland extreme arid and alpine climates, harbors endemic livestock gene pools with exceptional ecological adaptability. Among these, the Bayinbuluke sheep, a premium meat-and-fat dual-purpose endemic breed, has a history of natural evolution and directional breeding dating back to the late 18th century [6]. This breed thrives year-round in the Yulduz Basin of the central Tianshan Mountains. As a typical temperate arid subalpine steppe and alpine wetland ecosystem, its average elevation ranges from 2400 to 2600 m [7], with sub-zero annual average temperatures and a high-quality forage growing season of only about four months [8]. Extreme cold, hypoxia, and prolonged nutritional stress during the withered grass season impose severe natural selection pressures. As demonstrated by recent studies on plateau ruminants like Tibetan sheep, the skeletal muscle and lipid metabolic systems of these high-altitude breeds have undergone stringent natural selection under hypoxic and cold stress to survive in extreme environments [9,10,11]. In stark contrast, comparative evidence demonstrates that the Turpan Black sheep, which inhabits extremely hot and dry basins below sea level, has developed energy and nutrient accumulation patterns entirely distinct from the plateau phenotypes [11]. Selecting this breed provides a valid and extreme biological control within the same macro-geographical region. Furthermore, while macro-physiological adaptations such as enhanced lipid mobilization and polyunsaturated fatty acid (PUFA) enrichment are known survival strategies for plateau livestock, how these specifically enriched substrates translate into distinct volatile profiles during postmortem thermal processing remains a critical gap in food chemistry. This phenotypic divergence provides a unique natural biological comparative model [12] for exploring how environmental stress is associated with the reconstruction of muscle flavor metabolic networks in meat-producing animals [13].

Hypoxia and low temperatures represent the most prominent ecological stresses in high-altitude environments. To combat such environmental stressors, animals typically undergo profound reprogramming of their energy homeostasis and antioxidant defense systems at both the transcriptional and translational levels. For instance, accelerated lipid mobilization and the activation of sulfur-containing amino acid metabolism are crucial physiological strategies to mitigate cold stress and oxidative damage [14]. Our previous research has confirmed that high-altitude environments significantly remodel the lipid flux and energy metabolic networks in the skeletal muscle of Bayinbuluke sheep, triggering adaptive changes in physical meat quality phenotypes such as shear force and water-holding capacity [15]. However, from the perspective of microscopic food chemistry pathways, highly enriched PUFAs are highly susceptible to degradation into short-chain aldehydes, ketones, and esters with fresh and fruity notes via free radical chain reactions during thermal processing. Concurrently, sulfur-containing precursors like cysteine serve as core substrates in the Maillard reaction, generating crucial meaty heterocyclic compounds [16]. Nevertheless, how these adaptive metabolic remodelings occurring in vivo translate into volatile odor profiles dominated by Maillard reactions and lipid oxidative degradation during postmortem thermal processing remains a critical knowledge gap. Traditional single-target studies struggle to elucidate the complete biochemical cascade from macroscopic ecological adaptation to microscopic metabolic reprogramming, and ultimately to terminal sensory presentation.

Conventional meat flavor chemistry evaluation systems have long been constrained by an over-reliance on linear statistical screening based purely on concentration. This strictly mathematical dimension often masks core odor-active molecules that, despite their trace concentrations, have substantially crossed human olfactory perception thresholds. Therefore, returning to the fundamentals of sensory psychophysics and introducing a threshold-crossing evaluation system based on odor activity values (OAVs) is a highly informative approach for accurately mapping the divergent directions of flavor profiles in complex matrices [17]. Furthermore, given the highly nonlinear chemical transformations between raw meat precursors and cooked meat volatiles, traditional univariate linear mapping can no longer explain complex substrate competition mechanisms. It is urgently necessary to introduce multidimensional association networks and non-parametric rank correlation algorithms [18,19] to elevate the research dimension from isolated individual molecular analyses to global metabolic network spatial responses. Despite extensive research on high-altitude adaptation focusing on macroscopic physiological traits, a critical biological knowledge gap remains regarding how microscopic metabolic reprogramming in skeletal muscle alters the flavor precursor pool. We hypothesized that high-altitude adaptation is associated with altered intramuscular precursor pools and, consequently, distinct terminal volatile aroma profiles during thermal processing.

Building on this background, the present study utilizes Bayinbuluke and Turpan Black sheep as models. We first employ gas chromatography–mass spectrometry (GC-MS) combined with a threshold-crossing sensory evaluation strategy to precisely pinpoint the core odor-active compounds driving the flavor divergence between the two sheep groups. Recognizing that single-omics data are insufficient to bridge the massive bioinformatic gap from gene expression to final phenotypic presentation [20,21], this study deeply integrates multidimensional data from targeted flavor precursor metabolomics, transcriptomics, and proteomics. We investigate how high-altitude environments are associated with the remodeling of muscle lipid and water-soluble precursor pools via key regulatory enzymes and transporters. Finally, a Spearman rank correlation model is applied to construct a multidimensional association network linking biochemical precursor shifts to the global restructuring of volatiles. This study aims to break the limitations of single-omics and conventional statistical frameworks, providing systemic scientific support for elucidating the adaptive evolutionary mechanisms of livestock flavor quality in extreme geographical environments.

## 2. Materials and Methods

### 2.1. Experimental Animals and Sample Collection

Initially, 15 healthy 12-month-old male high-altitude Bayinbuluke sheep (BY group) and 15 low-altitude Turpan Black sheep (TLF group) were selected as experimental subjects. To strictly eliminate metabolic and physiological stress induced by long-distance transportation, all animals were slaughtered and sampled in situ at their respective native pastures. Simultaneously, to minimize batch effects introduced by environment and handling, the pre-slaughter treatment and sampling procedures at both locations were strictly executed by the identical research team. Due to the in situ observational design across distinct macro-geographical regions, the natural forage composition and diets of the two groups were inherently different and not artificially matched. This dietary disparity constitutes a primary environmental confounder associated with the altitudinal gradient. Prior to slaughter, all experimental animals were subjected to a strict 24 h fasting period, with water deprivation during the final 2 h. Post-slaughter, the longissimus dorsi muscle tissue located between the 12th and 13th thoracic vertebrae was rapidly collected. The samples were immediately snap-frozen in liquid nitrogen and subsequently transferred to a −80 °C ultra-low temperature freezer for future use.

For targeted absolute quantification (e.g., total fatty acids and specific water-soluble precursors), all biological replicates successfully passed instrumental quality control (n = 15 per group). However, during the global volatilomics data quality control phase, absolute sample pairing across different omics dimensions was required for the in-depth joint analysis. Individual samples were rigorously excluded based on a priori criteria: (1) internal standard recovery rates falling outside the 80–120% range in instrumental analysis, or (2) missing value rates exceeding 20% in global feature extraction. Based on these strict thresholds, incomplete sample pairs were excluded to prevent the introduction of statistical artifacts. Consequently, the actual effective biological replicates ultimately included in the global volatilomics and the subsequent multidimensional correlation network were 13 for the TLF group and 11 for the BY group. An a priori power analysis indicated that n ≥ 11 per group maintains a statistical power of >80% to detect a fold change of 1.5 at a significance level of 0.05, thereby ensuring the robustness of the downstream integration.

### 2.2. Volatilomics Determination and Sensory OAV Evaluation System

The extraction and analysis of muscle volatile flavor compounds were performed using a gas chromatography–mass spectrometry system (Agilent 8890-5977B, Agilent Technologies, Santa Clara, CA, USA) equipped with an olfactory port, coupled with solid-phase microextraction (SPME) technology. After removing connective tissue and visible fat from the meat surface, the samples were minced at low temperatures. Precisely 2.000 g of the meat sample was weighed into a 20 mL headspace vial, immediately followed by the addition of 1.0 μL of 2-methyl-3-heptanone (0.62 μg/μL) as an internal standard, and then hermetically sealed with a crimp cap. The sample was equilibrated at room temperature for 20 min before inserting a 50/30 μm DVB/CAR/PDMS extraction fiber (Supelco, Bellefonte, PA, USA) for headspace enrichment for 40 min. Upon completion of the extraction, the fiber was rapidly introduced into the GC inlet for thermal desorption at 250 °C for 5 min. To ensure analytical precision, each biological replicate was independently analyzed in technical triplicates.

Chromatographic separation was achieved using a DB-WAX polar capillary column (Agilent Technologies, Santa Clara, CA, USA, 30 m × 0.25 mm × 0.25 μm), with ultra-high purity helium (99.99%) as the carrier gas at a constant flow rate of 1.0 mL/min. The oven temperature programming was as follows: initial temperature at 40 °C held for 3.0 min, ramped to 200 °C at a rate of 5 °C/min, and subsequently increased to 230 °C at 5 °C/min and held for 3.0 min. The mass spectrometry system utilized an electron ionization source with an energy of 70 eV. The ion source and transfer line temperatures were set to 230 °C and 240 °C, respectively. The split ratio between the mass spectrometer detector and the olfactory port was set to 5:1, and the full-scan mass range was 50–400 *m*/*z*. It must be explicitly clarified that although the instrument was equipped with an olfactory port, no human sensory sniffing panel (olfactometry) was utilized in this study. Qualitative analysis was performed by rigorously comparing the full-scan mass spectra against the NIST 20.0 mass spectral library, retaining only identifications with a match score exceeding 80%. Quantitative analysis involved calculating absolute concentrations based on the peak area ratio of the target compounds to the internal standard.

To evaluate the actual sensory contribution of volatile compounds to the final meat flavor, this study incorporated the OAV evaluation system. The OAV is defined as the ratio of the absolute quantified concentration of a compound to its human olfactory perception threshold in an aqueous medium. An OAV ≥ 1 indicates that the compound has surpassed the olfactory threshold, making a substantial sensory contribution to the overall flavor profile; conversely, an OAV < 1 indicates that the flavor attribute is perceptually suppressed. Olfactory threshold data were primarily sourced from authoritative flavor chemistry compilation literature [22]. It must be acknowledged that utilizing olfactory perception thresholds derived from aqueous media may not perfectly represent the complex release dynamics within a solid meat matrix; however, the OAV system remains a robust and standardized chemical proxy for estimating relative sensory contributions.

### 2.3. Directed Mining of Flavor Precursor Pools

The extraction and quantification of total fatty acids (TFAs) were performed using Soxhlet extraction combined with gas chromatography. Precisely 2 to 5 g of meat sample, pre-dried to a constant weight at 105 °C, was weighed, placed in a filter paper thimble, and inserted into a Soxhlet extractor. Approximately 150 mL of petroleum ether (boiling range 30–60 °C) was added, and reflux extraction was conducted in a 70 °C water bath for 6 to 8 h. The solvent was recovered and dried to a constant weight to yield crude fat. Subsequently, 50 to 100 mg of the extracted lipid was accurately weighed into a test tube and dissolved in 4 mL of n-hexane. Then, 2 mL of 0.5 mol/L sodium hydroxide in methanol was added, and saponification was carried out in a 60 °C water bath for 30 min. After the reaction mixture cooled, 3 mL of 14% boron trifluoride in methanol was added for methylation at 60 °C for 10 min. Finally, 2 mL of n-hexane and an appropriate amount of saturated sodium chloride solution were added for vortex extraction. The supernatant, comprising the fatty acid methyl esters (FAMEs), was collected for instrumental analysis.

Fatty acid detection utilized a polar HP-88 column (Agilent Technologies, Santa Clara, CA, USA, 100 m × 0.25 mm × 0.20 μm), with the inlet and detector temperatures set at 250 °C and 260 °C, respectively. The temperature programming was as follows: initial 150 °C held for 1 min, ramped to 200 °C at 5 °C/min and held for 10 min, then increased to 230 °C at 2 °C/min and held for 5 min, using high-purity nitrogen as the carrier gas.

To capture a comprehensive metabolic landscape, abundance data for aqueous flavor precursors, specifically amino acids and monosaccharide derivatives, were acquired utilizing an untargeted liquid chromatography tandem mass spectrometry (LC-MS/MS) platform. The raw mass spectrometry data were converted to mzML format using ProteoWizard (version 3.0) and processed via XCMS(version 3.12.0) for peak extraction, alignment, and retention time correction. Features with a missing rate > 50% across samples were filtered out. Missing values were imputed using a K-Nearest Neighbors (KNN) algorithm (for missing rates < 50%) or replaced by 1/5 of the minimum value (for missing rates > 50%). Peak areas were normalized using the Support Vector Regression (SVR) method. Metabolite annotation was performed by cross-referencing an in-house laboratory database, integrated public and predictive databases, and the metDNA algorithm. Metabolites were retained only if they possessed a comprehensive annotation score > 0.5 and a Quality Control (QC) coefficient of variation (CV) < 0.3. Positive and negative ion modes were merged by retaining features with the highest qualitative grade and minimal CV. Following this global data validation, a hypothesis-driven “flavor-guided” extraction strategy was implemented. Rather than blind screening, water-soluble compounds structurally essential for postmortem flavor formation were exclusively extracted from the validated dataset: (1) free amino acids, focusing on sulfur-containing (e.g., L-cysteine) and branched-chain/aromatic amino acids; (2) reducing sugars and intermediates serving as carbon skeletons for the Maillard reaction; and (3) specific flavor-active dipeptides. Predefined precursors meeting these structural criteria and exhibiting significant inter-group variations (*p* < 0.05) were advanced for downstream multi-omics integration.

### 2.4. Transcriptomics and Proteomics Determination

The determination of the underlying molecular regulatory network in muscle tissues followed the multi-omics workflow previously established by our team [15]. Briefly, after total RNA extraction and purity validation, paired-end sequencing (2 × 150 bp) was performed using the Illumina NovaSeq 6000 (Illumina, San Diego, CA, USA) high-throughput platform. The filtered high-quality data were subsequently mapped to the sheep reference genome (Ovis aries ARS-UI_Ramb_v3.0). For proteomics analysis, total muscle protein was extracted, subjected to trypsin digestion, and desalted. Precise quantitative analysis of the peptides was then conducted using a Vanquish Neo nano-liquid chromatography system (Thermo Fisher Scientific, Waltham, MA, USA) coupled with an Orbitrap Astral high-resolution mass spectrometer (Thermo Fisher Scientific, Waltham, MA, USA) in data-independent acquisition (DIA) mode. Differential expression thresholds and bioinformatics parameters were strictly defined for both omics layers. For transcriptomics, differentially expressed genes (DEGs) were robustly identified using a fold-change (FC) ≥ 2 and a false discovery rate (FDR) < 0.01. For proteomics, the raw DIA mass spectrometry data were parsed using DIA-NN (v1.8.1) in a library-free mode, utilizing deep-learning parameters to predict a spectral library and enabling Match Between Runs (MBR) for qualitative and quantitative analysis. The data were searched against the UniProt Ovis aries database (uniprotkb_proteome_UP000002356_sheep_2024_09_25.fasta, comprising 23,108 sequences). Identifications were strictly filtered at a 1% FDR at both the precursor ion and protein levels. Differentially expressed proteins (DEPs) were defined by a *p*-value ≤ 0.05 and an FC ≥ 1.5 or ≤0.6667. To transparently delineate data reuse from novel findings, it is noted that the raw transcriptomic and proteomic sequence data have been previously deposited in public repositories (NCBI: PRJNA1306583; iProX: IPX0012591000) for a study focusing on macroscopic physical meat quality traits. However, the present investigation is entirely independent in its biological rationale. The lipid metabolism and sulfur-containing amino acid regulatory subnetworks were physically isolated from these repositories and integrated with newly generated GC-O-MS volatilomics and targeted flavor precursor datasets. This targeted re-mining strictly shifts the analytical dimension from physiological phenotypes to molecular flavor chemistry. It should be noted that while the raw transcriptomic and proteomic datasets were generated in our previous study to evaluate macrophysiological adaptations [15], the bioinformatics pipeline in the current work was entirely redesigned from a distinct food flavor chemistry perspective to mine novel metabolic hubs.

### 2.5. Data Processing and Statistical Analysis

Experimental data cleaning and visualization were performed in the R environment (version 4.3.3). Prior to statistical testing, omics data (metabolomics and volatilomics) were subjected to total ion current (TIC) and internal standard normalization to eliminate systematic analytical variances. Normal distribution was assessed using the Shapiro–Wilk test. Welch’s *t*-test or the non-parametric Mann–Whitney U test was employed for inter-group comparisons based on data distribution. To rigorously control false positives across high-dimensional multi-omics platforms, *p*-values of all univariate tests were subjected to multiple testing correction using the Benjamini–Hochberg method, outputting the false discovery rate (FDR).

To evaluate potential links between precursor pool remodeling and the terminal volatile profile, non-parametric Spearman rank correlation analysis was utilized. The calculation of the correlation coefficients (r) and significance testing matrices (*p*-values), along with the visualization of dual-color clustered heatmaps, was implemented using the corrplot package in R. All statistical significance thresholds were strictly set at *p* < 0.05. It should be noted that this correlation network serves as an exploratory statistical model to map spatial metabolic associations and highlight metabolic bottlenecks, rather than constituting definitive evidence of direct mechanistic or causal regulation. The effective sample size utilized for the correlation network was the strict intersection of samples passing quality control across all omics layers simultaneously (n = 24 in total; BY = 11, TLF = 13).

## 3. Results

### 3.1. Volatilomic Landscape and Threshold-Crossing Divergence of Key Odor-Active Compounds

To assess the divergent directions of meat flavor between low-altitude TLF and high-altitude BY sheep, 56 volatile compounds were initially identified across both groups. Constrained by the immense basal metabolic variance among individual animals, conventional linear differential screening models are prone to masking trace odor molecules that offer critical sensory contributions. Therefore, returning to the essence of sensory psychophysics, this study employed a sensory-guided filtering strategy. Initially, compounds were evaluated based on their variable importance in projection (VIP > 1). Subsequently, to isolate compounds with a direct impact on human perception, a strict sensory threshold filter was applied. Only compounds demonstrating an Odor Activity Value (OAV) ≥ 1 in at least one sheep group were retained as “core odor-active compounds” (Table 1). Although the absolute concentrations of some of these key compounds did not exhibit strict statistical significance (*p* > 0.05) due to high biological variance, their threshold-crossing behavior is sensorily highly relevant. Sub-threshold compounds (OAV ≪ 1 in both groups), despite their chemical presence and high VIP scores, provide no direct sensory contribution to the overall meat flavor and were thus categorized separately in the comprehensive dataset (Supplementary Table S1).

The comprehensive OAV assessment revealed that acetoin (OAV > 57), nonanal (OAV > 20), and 2,3-butanedione (OAV > 10) maintain high activity in both sheep meats. This consistent presence of high-activity compounds suggests that, regardless of altitude, both meats inherently possess a baseline characterized by milky and lipid-derived aroma attributes.

**Figure 1:**
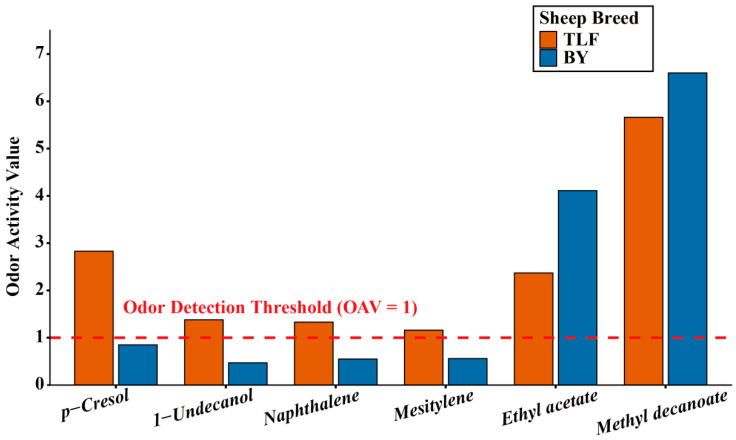
Switching and amplification effects of high-altitude adaptation on key odor-active compounds in the longissimus dorsi muscle. Grouped bar charts illustrate the OAV of six core differential volatile compounds between low-altitude TLF and high-altitude BY sheep. The horizontal red dashed line represents the human olfactory perception threshold (OAV = 1). Bars exceeding this threshold denote a perceivable, substantial contribution to the overall aroma profile, whereas those falling below indicate that the corresponding heavy basal flavor attributes are perceptually suppressed at the sensory level.

Building upon this baseline, altitude is associated with the fine-scale divergence of characteristic aroma profiles (Figure 1). In the low-altitude TLF meat, the OAV of p-cresol reaches 2.83, accompanied by 1-undecanol, naphthalene, and mesitylene crossing the olfactory threshold (OAV > 1). The moderate synergy of these compounds contributes to a recognizable classic meat aroma for TLF sheep. However, in the high-altitude BY meat, the concentrations of these four compounds—which are typically associated with heavy lipid and animal basal notes—undergo attenuation (FC < 0.50). Crucially, their OAVs all drop below the human olfactory perception threshold (OAV < 1). Even if the absolute concentration differences lack statistical significance, this targeted threshold-crossing shift indicates that the high-altitude phenotype is associated with perceptual suppression of these basal notes at the sensory level.

Coinciding with the perceptual suppression of these basal notes, BY meat presents a distinct volatile profile. The absolute concentration of ethyl acetate, which is associated with fruity and lipid-derived aroma notes, exhibits an increase (FC = 1.73) in the BY group, driving its OAV from 2.37 in TLF sheep up to 4.12. Concurrently, methyl decanoate increases from an OAV of 5.66 to 6.60. Synthesizing this bidirectional trend suggests that the sensory characteristics of high-altitude Bayinbuluke sheep meat are not simply a linear superposition of aroma compounds. Instead, this compositional shift is associated with an aroma profile characterized by reduced animal-like basal notes below the olfactory threshold and an amplified contribution of highly active ester compounds.

### 3.2. Landscape of the High-Altitude Remodeled Flavor Precursor Pool

**Figure 2:**
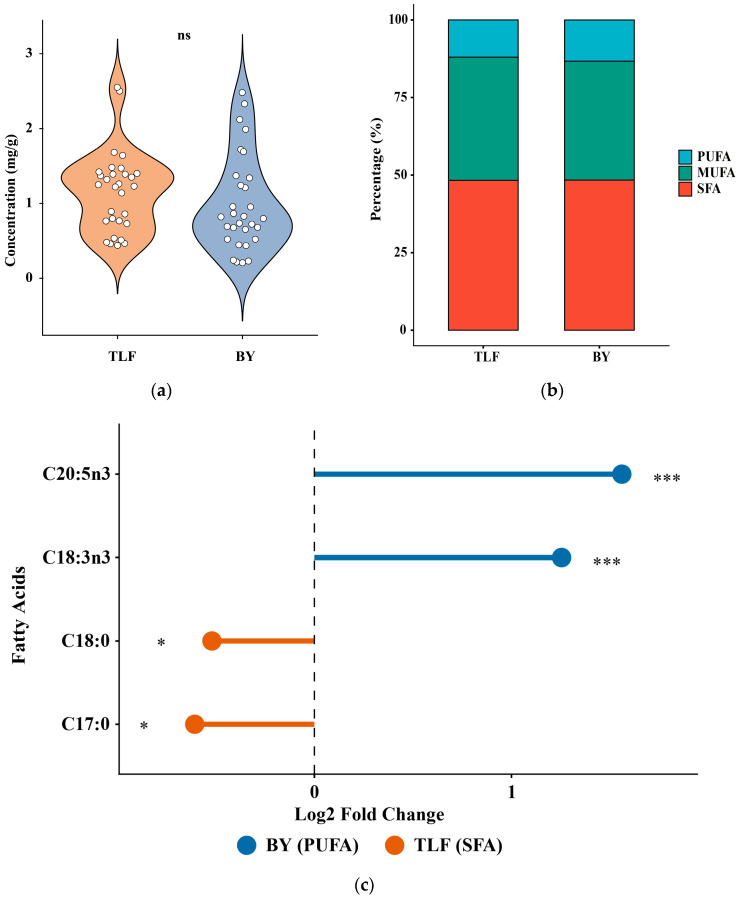

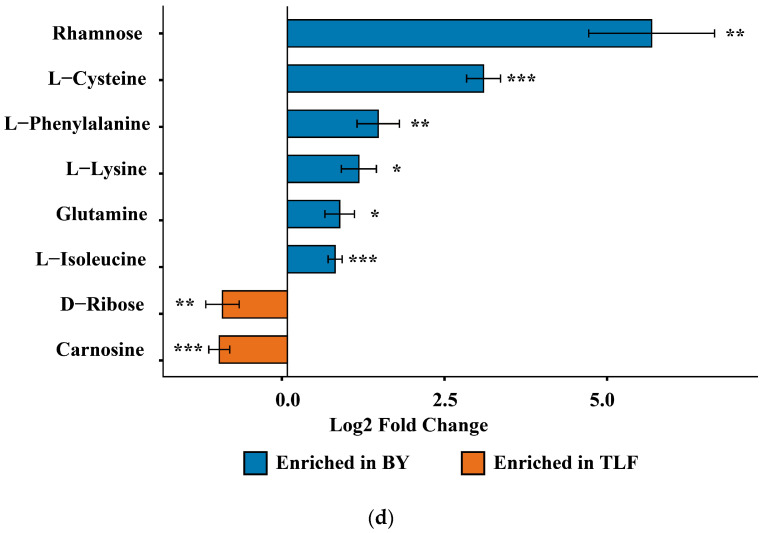
High-altitude-associated targeted remodeling of the flavor precursor pool in the longissimus dorsi muscle (n = 15 per group). (**a**) Comparison of total fatty acid concentrations between TLF and BY sheep, utilizing a violin plot combined with individual data points to display data density and individual variability. ns indicates no significant difference. (**b**) Stacked bar chart illustrating the relative percentages of polyunsaturated (PUFA), monounsaturated (MUFA), and saturated fatty acids (SFA), demonstrating a highly conserved macroscopic lipid class structure between the two groups. (**c**) Divergent dumbbell plot revealing the profound microscopic remodeling of targeted core FA precursors. Blue denotes significant enrichment in BY sheep (PUFAs), whereas orange denotes enrichment in TLF sheep (SFAs). (**d**) Divergent bar chart visualizing the Log2 Fold Change (BY/TLF) of water-soluble Maillard reaction precursors, including core amino acids, carbohydrates, and dipeptides. Error bars represent the standard error of the mean (SEM). Blue bars (positive values) indicate significant enrichment in the high-altitude BY sheep, whereas orange bars (negative values) indicate accumulation in the low-altitude TLF sheep. ns indicates no significant difference; *, **, and *** represent *p* < 0.05, *p* < 0.01, and *p* < 0.001, respectively. Abbreviations: TLF, low-altitude Turpan Black sheep; BY, high-altitude Bayinbuluke sheep; SFAs, saturated fatty acids; MUFAs, monounsaturated fatty acids; PUFAs, polyunsaturated fatty acids; FA, fatty acid.

Intramuscular fatty acids serve as the core substrates generating characteristic lipid aromas during the thermal degradation of meat. Figure 2a shows no significant statistical difference in total fatty acid concentration within the longissimus dorsi muscle between the low-altitude TLF and high-altitude BY sheep. Further group proportion analysis (Figure 2b) indicates that the macroscopic lipid composition ratios (SFA, MUFA, and PUFA) remained relatively stable across both meats, reflecting the high environmental conservation of the basal lipid metabolic network in skeletal muscle. However, beneath the facade of this relatively stable macroscopic baseline, characteristic precursor molecules with high reactivity underwent profound microscopic remodeling (Figure 2c). Targeted core precursor analysis revealed that saturated fractions associated with traditional heavy basal and oxidative notes, such as heptadecanoic acid (C17:0) and stearic acid (C18:0), accumulated significantly in the TLF group (*p* < 0.05). Conversely, core members of the omega-3 family, including α-linolenic acid (C18:3n3) and eicosapentaenoic acid (EPA, C20:5n3), exhibited highly significant targeted enrichment in the BY group (*p* < 0.001). These omega-3 PUFAs are highly susceptible to lipid oxidative degradation and are plausibly linked to the generation of fruity and lipid-derived aroma notes. This precursor shift pattern, combining macroscopic conservation with targeted surges, without altering the total muscle lipid load, establishes a material foundation with reactive potential hypothesized to contribute to the characteristic lipid aroma of high-altitude sheep meat.

Beyond the lipid degradation pathway, the Maillard water-soluble precursor pool, which dictates the trajectory of thermal reaction pathways, similarly underwent a precise targeted shift (Figure 2d). Divergent bar chart results indicated that L-cysteine, a key substrate convertible into core meat aromas (sulfur-containing heterocycles), presented highly significant enrichment in the BY group (*p* < 0.001). Simultaneously, aromatic and branched-chain amino acids (L-phenylalanine, L-isoleucine) that impart complex aroma profiles, alongside umami amino acids (glutamine), were significantly upregulated in the BY group. Regarding carbohydrate participants, rhamnose, which possesses an intense sweet flavor and furan-aroma synthesis potential, accumulated substantially in the BY group. In contrast, the relative abundances of D-ribose and the dipeptide carnosine, which exhibit extremely high Maillard reactivity and are often associated with the rapid generation of advanced, sometimes undesirable, Maillard products, decreased significantly. This directional enrichment and depletion profile of water-soluble precursors suggests that the high-altitude-associated phenotype may suppress certain off-flavor pathways while intensifying the synthetic potential for fruity and sweet flavor profiles at the substrate level.

### 3.3. Multi-Omics Integration Reveals the Core Metabolic Network Associated with the Targeted Remodeling of Flavor Precursors

To explore the underlying molecular mechanisms potentially linking high-altitude environments to structural shifts in the flavor precursor pool of the longissimus dorsi muscle in Bayinbuluke sheep, this study integrated transcriptomic and proteomic data for targeted mining. The results demonstrate that high-altitude adaptation coincides with the reprogramming of lipid flux and amino acid metabolism at both the transcriptional and translational levels; relevant targets are summarized in Table 2.

Within the lipid metabolic regulatory network, multi-omics data revealed a synergistic effect potentially inhibiting fat accumulation and promoting lipid mobilization. Transcriptomic results showed a significant downregulation of *GPAT3*, a key rate-limiting enzyme gene catalyzing the initial step of triglyceride synthesis. Simultaneously, proteomic data indicated a significant decrease in the protein expression of APOC3, a critical inhibitor of lipoprotein lipase (LPL) (log2FC = −0.82, *p* < 0.01). The transcriptional suppression of *GPAT3* theoretically blocks the conversion of fatty acid substrates into inert lipid droplets, whereas the reduced protein abundance of APOC3 releases the restriction on lipolysis. This cross-omics bidirectional regulatory pattern suggests a potential mechanism by which the fatty acid pool of Bayinbuluke sheep meat (Figure 2) achieves the targeted accumulation of highly active PUFAs without increasing the total lipid load.

In the amino acid flavor precursor metabolic pathway, the combination of synthesis activation and degradation blockade is associated with the enrichment of characteristic substrates. At the transcriptional level, *CTH*, the core enzyme gene directly catalyzing the generation of L-cysteine in the transsulfuration pathway, was significantly upregulated (FDR < 0.01). This closely corresponds to the targeted enrichment of water-soluble precursors in Figure 2d, providing a robust material basis for generating sulfur-containing active volatiles. At the protein level, the expression of acetoacetyl–CoA synthetase (AACS), which participates in the branched-chain amino acid degradation pathway, underwent a sharp downregulation (log2FC = −1.31, *p* < 0.05). The low expression of the AACS protein is hypothesized to decelerate the consumption rate of branched-chain amino acids, such as isoleucine, in skeletal muscle, plausibly contributing to the significant accumulation of these precursors for volatile fruity and sweet aromas. Synthesis of the multi-omics evidence indicates that the high-altitude-associated phenotype, by systematically reconstructing the metabolic enzyme and transporter networks, creates a biochemical environment conducive to the generation of specific flavor precursors.

### 3.4. Multi-Omics Association Network Reveals the Multidimensional Regulation of Terminal Flavor Profiles by Precursor Shifts

**Figure 3:**
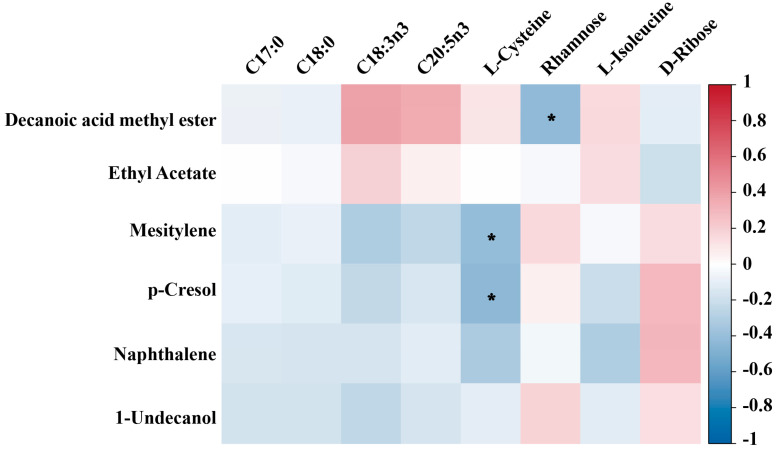
Spearman rank correlation heatmap between targeted flavor precursors and key odor-active volatiles (n = 24). The color scale represents the correlation coefficient (r), ranging from dark blue (strong negative correlation, r = −1) to dark red (strong positive correlation, r = 1). * indicates *p* < 0.05.

Given that the high-altitude environment is associated with profound remodeling of the metabolic enzyme expression profile and the flavor precursor pool, this study further employed Spearman rank correlation analysis to construct an association mapping network between core precursors and key odor-active compounds (Figure 3). To avoid introducing statistical noise and obscuring the core metabolic network, this correlation analysis intentionally focused on a highly refined subset rather than all identified compounds. These selected molecules represent the exact differential markers that successfully passed the rigorous filtering strategies described in previous sections. The aim here is to explore the potential regulatory associations between structural precursor shifts and terminal characteristic flavors. Considering the nonlinear nature of precursor-to-volatile conversion during meat heating, the rank correlation model more robustly captures association trends within complex matrices.

The correlation heatmap clearly delineates the bidirectional pathways of flavor molecules. In the sensory suppression pathway, targeted water-soluble precursors demonstrate a robust remodeling capacity. L-cysteine, a core Maillard reaction substrate highly enriched in Bayinbuluke sheep, exhibited significant negative correlations with p-cresol and mesitylene, compounds associated with heavy animalic and basal notes in Turpan sheep (r = −0.43, *p* = 0.035; r = −0.41, *p* = 0.048). This suggests that the targeted enrichment of sulfur-containing precursors likely alters the competitive pathways of thermal degradation reactions, thereby contributing to the sub-threshold perceptual suppression of animal off-flavors.

In the ester-derived aroma generation pathway, α-linolenic acid (C18:3n3), a highly active polyunsaturated fatty acid heavily accumulated in BY sheep, presented a non-significant moderate trend (r = 0.39, *p* = 0.058) with methyl decanoate, which is associated with fruity and lipid-derived aromas. Synthesizing this multidimensional evidence chain suggests that high-altitude adaptation, by targeted reconstruction of the intramuscular FA pool and water-soluble precursor pool, establishes a material foundation that favors the perceptual suppression of undesirable basal notes and the selective retention of highly active ester compounds during the final thermal presentation.

## 4. Discussion

The formation of meat flavor is an exceedingly complex, nonlinear thermal biochemical process. Through joint multi-omics analysis, this study explores the potential associations between high-altitude environments and sheep meat flavor quality. The research suggests that high-altitude adaptation coincides with a directional restructuring of the lipid- and water-soluble precursor pools in muscle tissue via targeted regulation at the transcriptional and translational levels [27,28]. This structural shift at the substrate level crosses the human olfactory perception threshold during meat heating, ultimately contributing to the divergence of the meat flavor phenotype observed in Bayinbuluke sheep.

Using a comprehensive OAV assessment, this study confirmed that both groups of sheep meat retain a consistent aroma baseline centered on acetoin and nonanal [29], which primarily originate from the thermal oxidation of common fatty acids [30]. Upon this baseline, the high-altitude-associated phenotype exhibits a perceptual suppression effect. Compounds like p-cresol and naphthalene, which impart heavy animal basal notes in low-altitude meat, undergo substantial attenuation and fall below the olfactory perception threshold in high-altitude meat [31,32,33]. Since p-cresol typically originates from the anaerobic fermentative degradation of aromatic amino acids by rumen microbiota [33,34], its significant reduction is likely related to shifts in pasture vegetation and host metabolic clearance capacity. This sensory suppression of animalic notes and the targeted amplification of ester-derived aromas establish the chemical foundation for the distinct sensory phenotype of Bayinbuluke sheep meat.

The fatty acid composition of muscle lipids serves as the core substrate pool shaping characteristic lipid aromas. While maintaining macroscopic lipid balance, high-altitude sheep meat achieves a significant enrichment of highly reactive PUFAs, such as EPA. Multi-omics integration suggests that this remodeling may be linked to the bidirectional restriction of lipid metabolic pathways: the transcriptional downregulation of *GPAT3* and the translational suppression of APOC3. *GPAT3* dominates the conversion of fatty acids into neutral triglycerides [35]; its impeded expression is hypothesized to restrict excessive esterification at the biosynthetic source [15]. Concurrently, APOC3 is a key endogenous inhibitor of LPL [36]; its downregulation effectively releases this restriction, promoting targeted lipid uptake. From an evolutionary perspective, this PUFA enrichment strategy represents a metabolic adjustment to maintain cell membrane fluidity under cold and hypoxic environments [27,37]. This specific lipid composition becomes a highly reactive flavor substrate source during postmortem thermal processing, generating short-chain aldehydes, ketones, and ester derivatives characterized by fresh and fruity notes [38,39].

Beyond the lipid pathway, the structural reconstruction of the Maillard reaction precursor pool further dictates the flavor evolution. Multi-omics data revealed that the expansion of the sulfur-containing amino acid pool is associated with the upregulated expression of *CTH*. Since cysteine is the absolute rate-limiting precursor for synthesizing glutathione, the upregulation of *CTH* likely acts as a molecular defense mechanism against environmental oxidative stress [40,41]. During thermal processing, these enriched amino acids synergize with rhamnose to participate in the Maillard reaction [42]. Correlation analysis revealed a significant negative relationship between precursors like L-cysteine and compounds like p-cresol, suggesting that specific substrate ratio may intervenes in competitive thermal degradation pathways, thereby inhibiting the generation of potential undesirable intermediates. Simultaneously, the downregulation of AACS is hypothesized to preserves the substrate reserve of isoleucine and other BCAAs, which are efficiently converted into branched-chain derivatives via Strecker degradation [16,43].

Notably, the targeted reconstruction of the skeletal muscle flavor precursor pool in Bayinbuluke sheep highly aligns with the biochemical laws of convergent evolution in plateau mammals coping with extreme environments [44]. Joint multi-omics studies on yaks and Tibetan pigs have similarly confirmed that cold and hypoxia are associated with systemic lipid mobilization and PUFA enrichment [45], alongside compensatory activation of sulfur-containing amino acid metabolism [28]. The precursor shift trajectory observed in this study is consistent with these cross-species molecular evidences. This macroscopic biochemical commonality highlights a broader biological hypothesis: the specific lipid aroma and pure basal flavor of high-altitude meat may represent convergent metabolic adaptations associated with extreme ecological pressures.

Although this study constructed a logical framework of environment-associated flavor evolution from a systems biology dimension, several limitations inherent to the cross-sectional, cross-breed observational design must be explicitly acknowledged. First, a central limitation is the inherent “breed versus environment” confound. Because Bayinbuluke and Turpan Black sheep are genetically distinct breeds raised in radically different habitats, it is currently impossible to definitively parse whether the observed flavor precursor shifts are exclusively associated with altitude-induced stress (hypoxia and cold), breed-specific genetic backgrounds, or disparities in forage composition. Future multifactorial designs, such as common-garden feeding experiments or reciprocal transplant studies, are required to disentangle these variables. Second, comparing only two extreme altitude points limits our ability to delineate the continuous dynamic evolutionary trajectory of flavor precursors along an elevational gradient.

Furthermore, while the multi-omics correlation networks provide high-confidence statistical spatial mapping evidence and a plausible biological blueprint, these relationships remain fundamentally hypothetical. Without direct functional validation experiments, such as targeted gene knockout models in vivo or absolute quantitative verification utilizing stable isotope tracing combined with in vitro biomimetic meat matrix models, biochemical causality cannot be definitively established. Additionally, although the OAV approach offers a robust chemical proxy for sensory perception, the absence of evaluation by a trained sensory panel limits the direct psychophysical confirmation of the proposed flavor shifts. Finally, the concept of convergent evolution discussed herein should be interpreted as a broader biological hypothesis rather than as a definitive conclusion directly proven by this isolated dataset. Future studies must integrate these experimental validations to transition from metabolic associations to verified biochemical causation.

Nevertheless, the basal metabolic associations established in this study have illuminated a potential path for the high-value development of endemic livestock resources. The characteristic metabolic hub targets, such as *CTH* and *GPAT3*, possess substantial potential as critical biomarkers for breeding plateau-specific premium meat and for marker-assisted identification of flavor traits.

## 5. Conclusions

From the dual dimensions of ecological adaptation and microscopic metabolic reprogramming, this study comprehensively reveals the directional remodeling mechanisms of high-altitude environments on the characteristic flavor quality of sheep meat. The research confirms that high-altitude habitats are associated with the targeted enrichment of highly reactive polyunsaturated fatty acids in muscle tissue by downregulating the transcription of *GPAT3*, the rate-limiting enzyme gene for triglyceride synthesis, and suppressing the translation of APOC3, a lipolysis inhibitor. Concurrently, driven by underlying physiological defense requirements such as oxidative stress resistance, the significant upregulation of *CTH*, the core enzyme gene in the transsulfuration pathway, and the downregulation of AACS, a branched-chain amino acid degradation protein, synergistically drive the substantial accumulation of core water-soluble precursors like cysteine and rhamnose. Induced by extreme survival pressures, this structural shift in the skeletal muscle precursor pool exerts critical substrate competition and chemically antagonistic effects during postmortem thermal processing. Consequently, this structural shift is strongly associated with the directional amplification of premium ester compounds conferring delicate fruity aromas and the sub-threshold suppression of animal off-flavors, represented by p-cresol. In summary, the pure plateau flavor of Bayinbuluke sheep meat reflects a shared metabolic pattern associated with extreme ecological pressures. However, the exact mechanisms driving these changes remain a robust hypothesis requiring further validation. The limitations inherent to our cross-sectional comparative design highlight the need for future studies. Integrating stable isotope tracing, controlled feeding experiments, enzyme activity measurements, and formal sensory evaluations will be essential to transition from metabolic associations to a comprehensive, causal understanding of the biochemical mechanisms underlying flavor formation in high-altitude sheep meat.

## Figures and Tables

**Table 1:** Identification of key odor-active compounds associated with the sensory profiles of TLF and BY sheep meat based on OAV threshold assessment.

Compound	Conc. TLF (μg/kg)	Conc. BY (μg/kg)	*p*-Value	Odor Threshold (μg/kg)	OAV (TLF)	OAV (BY)	FC	Reference
Methyl decanoate	24.35 ± 11.10	28.39 ± 9.08	0.13	4.3	**5.66**	**6.6**	1.17	[23]
*p*-Cresol	11.02 ± 4.08	3.31 ± 0.78	0.18	3.9	**2.83**	0.85	0.3	[24]
Ethyl Acetate	11.86 ± 2.18	20.58 ± 7.19	0.27	5	**2.37**	**4.12**	1.73	[25]
1-Undecanol	118.36 ± 54.86	40.28 ± 12.35	0.91	86	**1.38**	0.47	0.34	[23]
Naphthalene	6.64 ± 2.34	2.73 ± 0.55	0.49	5	**1.33**	0.55	0.41	[26]
Mesitylene	3.49 ± 1.00	1.7 ± 0.27	0.09	3	**1.16**	0.57	0.49	[22]

Note: Conc. represents the mean absolute concentration ± standard error of the mean (SEM). Statistical significance between groups was determined using the appropriate univariate test (*p* < 0.05). FC indicates the ratio of the mean concentration in BY to TLF. OAV is calculated as the ratio of concentration to the odor threshold. OAVs ≥ 1.0 are highlighted in bold, indicating an active and perceivable contribution to the overall flavor profile. Odor threshold values (in aqueous medium) were derived from the authoritative compilation by Van Gemert (2011) [22]. Where primary sources were not independently accessible, data were referenced via the compilation [22].

**Table 2:** Core regulatory genes and proteins associated with the targeted remodeling of flavor precursors in the longissimus dorsi muscle revealed by multi-omics integration.

Symbol	Omics Level	KEGG Pathway	Mean Expression (TLF)	Mean Expression (BY)	Log2FC	Significance	Regulation
*GPAT3*	Transcriptome	Glycerolipid metabolism	7.80	5.04	−0.81	FDR = 0.003	Down
APOC3	Proteome	PPAR signaling pathway/Lipid transport	0.41	0.23	−0.82	*p* = 0.002	Down
*CTH*	Transcriptome	Cysteine and methionine metabolism	1.09	2.12	0.74	FDR = 0.009	Up
AACS	Proteome	Valine, leucine and isoleucine degradation	0.19	0.08	−1.31	*p* = 0.016	Down

Note: Italicized text denotes genes (transcriptomics), whereas non-italicized text denotes proteins (proteomics). Mean Expression represents the average FPKM (Fragments Per Kilobase of transcript per Million mapped reads) values for genes and the average relative abundance for proteins. Log2FC represents the relative expression levels in the high-altitude BY group compared to the low-altitude TLF group. Positive values indicate upregulation in the BY group, whereas negative values indicate downregulation. Statistical significance is expressed as the FDR for transcriptomic data and as the *p*-value for proteomic data.

## Data Availability

The raw transcriptomic and proteomic data supporting the secondary targeted flavor precursor mining in this study are openly available at the National Center for Biotechnology Information (NCBI) under BioProject PRJNA1306583 and the Integrated Proteome Resources (iProX) under accession number IPX0012591000, respectively. The metabolomic and additional supplementary data are available in Figshare at http://doi.org/10.6084/m9.figshare.29551862. These core datasets were originally generated and thoroughly described by our research group in a previous physiological study, and have been re-analyzed in the current work from the distinct perspective of food flavor chemistry.

## Supplementary Materials

The following supporting information can be downloaded at https://www.mdpi.com/article/10.3390/foods15142515/s1: Table S1: Quantitative profiles and OAVs of volatile compounds identified in the longissimus dorsi muscle of TLF and BY sheep. Reference [46] is cited in the supplementary materials.

## Abbreviations

The following abbreviations are used in this manuscript.

PUFA	Polyunsaturated fatty acid
MUFA	Monounsaturated fatty acid
SFA	Saturated fatty acid
OAV	Odor activity value
GC-O-MS	Gas chromatography–olfactometry–mass spectrometry
TFA	Total fatty acids
FAME	Fatty acid methyl esters
LC-MS/MS	Liquid chromatography tandem mass spectrometry
NCBI	National Center for Biotechnology Information
iProX	Integrated Proteome Resources
FDR	False discovery rate
FC	Fold change
EPA	Eicosapentaenoic acid
