# Supplementary material for: Alpine Meadow Habitat Is Associated with Characteristic Flavor Formation in Bayinbuluke Sheep Meat Through Metabolic Reprogramming
Source: Foods. 2026 Jul 16;15(14):2515. doi: 10.3390/foods15142515 (PMC13407686; doi:10.3390/foods15142515)
Supplement: Supplementary file 1 [file foods-15-02515-s001.zip › foods-4383702-supplementary.pdf]

Table S1. Quantitative profiles and OAV of volatile compounds identified in the longissimus dorsi muscle of TLF and BY sheep

**Compound**	**Conc. TLF (μg/kg)**	**Conc. BY (μg/kg)**	**Odor Threshold (μg/kg)**	**OAV (TLF)**	**OAV (BY)**	**FC**	**Reference**
Acetoin	973.05	806.38	14	**69.5**	**57.6**	0.83	[22] (Ref: Boonbumrung et al, 2001)
Nonanal	27.10	22.99	1.1	**24.64**	**20.9**	0.85	[22] (Ref: Giri et al, 2010)
2,3-Butanedione	82.84	61.76	6	**13.81**	**10.29**	0.75	[22] (Ref: Leksrisompong, 2008; Leksrisompong et al, 2010)
Methylamine, N,N-dimethyl-	64.18	40.08	8	**8.02**	**5.01**	0.62	[22] (Ref: Tuorila et al, 1982)
Methyl decanoate	24.35	28.39	4.3	**5.66**	**6.6**	1.17	[22] (Ref: Schnabel et al, 1988)
p-Cresol	11.02	3.31	3.9	**2.83**	0.85	0.30	[22] (Ref: Czerny et al, 2008)

Ethyl Acetate	11.86	20.58	5	**2.37**	**4.12**	1.73	[22] (Ref: Giri et al, 2010)
1-Undecanol	118.36	40.28	86	**1.38**	0.47	0.34	[22] (Ref: Schnabel et al, 1988)
Naphthalene	6.64	2.73	5	**1.33**	0.55	0.41	[22] (Ref: Zoeteman et al, 1971)
Mesitylene	3.49	1.70	3	**1.16**	0.57	0.49	[22] (Ref: De Grunt, 1975)
Nonanoic acid, methyl ester	64.70	54.83	60	**1.08**	0.91	0.85	Buttery et al., 1988 *
5-Nonanone	3.99	2.65	8.2	0.49	0.32	0.66	[22] (Ref: Schnabel et al, 1988)
Octanoic acid, methyl ester	72.89	44.23	200	0.36	0.22	0.61	[22] (Ref: Pino & Mesa, 2006)
Indole	8.17	8.40	21	0.39	0.40	1.03	[22] (Ref: Karaguil-Yuiceer et al, 2003)
Methanethiol	0.57	0.73	0.2	2.85	3.65	1.28	[22] (Ref: Milo &

							Grosch, 1997; Kerscher& Grosch, 2000)
Benzene	8.49	10.94	72	0.12	0.15	1.29	[22] (Ref: Alexander et al, 1982)
Benzaldehyde	91.31	165.93	750.89	0.12	0.22	1.82	[22] (Ref: Giri et al, 2010)
3-Heptanone	5.28	2.27	80	0.07	0.03	0.43	[22] (Ref: Schnabel et al, 1988)
Dodecanoic acid, methyl ester	36.87	19.32	NA	-	-	0.52	
Acetic acid, methyl ester	37.03	146.35	1500	0.02	0.1	3.95	[22] (Ref: Schnabel et al, 1988)
Butanoic acid, 2-methyl-	1.27	1.59	100	0.01	0.02	1.25	[22] (Ref: Gassenmeier& Schieberle, 1995)
Acetophenone	0.37	0.60	65	0.01	0.01	1.63	[22] (Ref: Buttery et al, 1988a; Buttery & Ling,

							1995)
2-Methylheptanoic acid	4.73	2.48	29500	0	0	0.52	[22] (Ref: Amoore, 1976)
3-Hexen-1-ol, (E)-	0.19	0.12	110	0	0	0.64	[22] (Ref: Tamura et al, 2001; Boonbumrung et al, 2001)
Pentanoic acid, 2-methyl-	2.80	2.12	10000	0	0	0.75	[22] (Ref: Amoore, 1976)
Hexanoic acid, methyl ester	8.24	2.42	70	0.12	0.03	0.29	[22] (Ref: Pino & Mesa, 2006)
Hexadecanoic acid, methyl ester	107.09	155.04	＞2000	< 0.054	< 0.078	1.45	[22] (Ref: Buttery et al, 1988a)
Methyl tetradecanoate	36.13	38.02	NA	-	-	1.05	
2H-Thiopyran, 3,4-dihydro-	0.31	0.13	NA	-	-	0.43	
2-Butenoic acid, methyl ester	1.83	1.11	NA	-	-	0.61	
3-Methyl-2-butenoic acid, cyclobutyl ester	1.11	0.48	NA	-	-	0.43	

N-Methyl-L-proline, pentyl ester	0.18	0.08	NA	-	-	0.48
2-Nonanone, 3-(hydroxymethyl)-	111.96	64.78	NA	-	-	0.58
Oxalic acid, butyl cyclobutyl ester	2.51	3.77	NA	-	-	1.50
3-Hydroxy-3-methyl-2-butanone oxime	0.22	0.08	NA	-	-	0.39
2-Methylhept-6-en-3-one	1.53	0.65	NA	-	-	0.43
Cycloheptanecarboxylic acid, 1-amino-	0.34	0.16	NA	-	-	0.47
Hexane, 2,4-dimethyl-	7.73	4.07	NA	-	-	0.53
Methyl glyoxal	4.33	2.87	NA	-	-	0.66
N-Isopropyl-3-phenylpropanamide	0.99	1.59	NA	-	-	1.60
Butane, 2-methyl-	16.89	23.15	NA	-	-	1.37
1-Pentanamine, N-methyl-	1.01	0.58	NA	-	-	0.57
Hydroxypivalic acid	5.95	2.17	NA	-	-	0.37
1-Pentanamine, N-ethyl-	0.56	1.42	NA	-	-	2.53
1-Hexanone, 5-methyl-1-phenyl-	2.89	1.51	NA	-	-	0.52
Ethanone, 1-oxiranyl-	8.16	3.32	NA	-	-	0.41

Cyclopropane, pentyl-	8.93	4.58	NA	-	-	0.51
Ethanone, 2-(formyloxy)-1-phenyl-	5.14	2.81	NA	-	-	0.55
N-Dodecyl-5-methyl-2-pyrrolidone	1.33	2.58	NA	-	-	1.93
1,14-Tetradecanediol	19.77	12.27	NA	-	-	0.62
N-.alpha.-Acetylglycinamide	1.57	1.68	NA	-	-	1.07
5-Aminovaleric acid	0.46	0.32	NA	-	-	0.70
2-Octen-1-ol, (Z)-	6.01	5.24	NA	-	-	0.87
2-Aminocyanoacetamide	1.45	0.93	NA	-	-	0.64
3-Amino-2,4-dimethylpentane	1.89	1.75	NA	-	-	0.93
9-Dodecenoic acid, methyl ester, (E)-	10.54	8.47	NA	-	-	0.80

Note: Conc. represents the mean absolute concentration. FC indicates the ratio of the mean concentration in the BY group to that in the TLF group. OAV is calculated as the ratio of the absolute concentration to its corresponding odor threshold. OAVs ≥ 1.0 are highlighted in bold, indicating a perceivable and substantial sensory contribution to the overall flavor profile. NA indicates that the olfactory threshold for the specific compound is not available in the current literature. Odor threshold values (in aqueous medium) were generally evaluated based on the compilation by Van Gemert (2011) [22]. References formatted as "[22] (Ref: Author, Year)" indicate the specific primary source indexed within this compilation. The threshold marked with an asterisk (*) was independently sourced from: Buttery, R. G., Turnbaugh, J. G., & Ling, L. C. (1988). Contribution of volatiles to rice aroma. Journal of Agricultural and Food Chemistry, 36(5), 1006–1009.
